# The *Lactobacillus casei* Group: History and Health Related Applications

**DOI:** 10.3389/fmicb.2018.02107

**Published:** 2018-09-10

**Authors:** Daragh Hill, Ivan Sugrue, Conor Tobin, Colin Hill, Catherine Stanton, R. Paul Ross

**Affiliations:** ^1^School of Food and Nutritional Sciences, University College Cork, Cork, Ireland; ^2^Teagasc, Moorepark, Food Research Centre, Fermoy, Ireland; ^3^APC Microbiome Ireland, Cork, Ireland; ^4^School of Microbiology, University College Cork, Cork, Ireland

**Keywords:** lactobacillus, casei, paracasei, rhamnosus, health, stress, taxonomy

## Abstract

The *Lactobacillus casei* group (LCG), composed of the closely related *Lactobacillus casei, Lactobacillus paracasei*, and *Lactobacillus rhamnosus* are some of the most widely researched and applied probiotic species of lactobacilli. The three species have been extensively studied, classified and reclassified due to their health promoting properties. Differentiation is often difficult by conventional phenotypic and genotypic methods and therefore new methods are being continually developed to distinguish the three closely related species. The group remain of interest as probiotics, and their use is widespread in industry. Much research has focused in recent years on their application for health promotion in treatment or prevention of a number of diseases and disorders. The LCG have the potential to be used prophylactically or therapeutically in diseases associated with a disturbance to the gut microbiota. The group have been extensively researched with regard to stress responses, which are crucial for their survival and therefore application as probiotics.

## Introduction

There are more than 200 species of *Lactobacillus*, the largest and most diverse genus within the lactic acid bacteria (LAB) (Sun et al., 2015). *Lactobacillus* spp. are part of the microbiota of humans and animals (Casey et al., 2004) where they colonize the gastrointestinal tract (GIT) and the urogenital tract (Parolin et al., 2015). They are also found in a variety of food products from fruit and vegetables (Savino et al., 2012) to a range of naturally fermented products (Ao et al., 2012; Owusu-Kwarteng et al., 2015). *Lactobacillus* spp. have been deployed and studied extensively as fermentation starter cultures (Aryana and Olson, 2017) and as probiotics (Ryan et al., 2015; Pace et al., 2015). Their long history of use in fermented products has led to their recognition as GRAS (generally recognized as safe) by the US Food and Drug Authority (FDA), and earned them a place on the QPS (qualified presumption of safety) list assembled by the European Food Safety Authority (EFSA, 2016).

The *Lactobacillus casei* group (LCG), comprised mainly of the closely related *Lactobacillus casei, Lactobacillus paracasei*, and *Lactobacillus rhamnosus* species, are among some of the most studied species due to their commercial, industrial and applied health potential. Commercially, they are used to ferment dairy products, often producing foods with improved flavor and texture. They have also been found to produce many bioactive metabolites which can confer host benefits when consumed (Dietrich et al., 2014). As such, many LCG strains are considered to be probiotics. One member, *L. rhamnosus* GG (LGG), is perhaps one of the most studied bacterial strains in relation to health applications (Segers and Lebeer, 2014).

This review will discuss the complex issues relating to the taxonomy of the LCG, current research and novel applications of species and strains within the group for health promotion, and will consider stress response mechanisms crucial for probiotic survival.

## A Brief Taxonomic History

The LCG is composed of three genotypically and phenotypically related facultatively heterofermentative species, *L. casei, L. paracasei*, and *L. rhamnosus.* The taxonomy of these species has been extensively investigated in the past, as many strains within the group are commonly applied as starter cultures and probiotics. As species of the LCG are of significant commercial value, their relationship to one another has been the focus of much research. It is important to emphasize that distinguishing them is important to ensure correct evaluation of the literature and to avoid confusion between related probiotic strains in food and health-associated products. However, the taxonomic history of the LCG has been both convoluted and challenging.

*Lactobacillus casei* was first proposed as a novel species in 1971 (Hansen and Lessel, 1971), but this characterisation was first questioned in 1996 (Dicks et al., 1996). It was proposed that the type strain *L. casei* ssp. *casei* ATCC 393 and *L. rhamnosus* ATCC 15820 should be reclassified as the previously described *L. zeae* (Kuznetsov, 1959), due to high similarity to one another and separation from the rest of the LCG cluster. The name *L. paracasei* was rejected, and *L. casei* ATCC 334 was suggested as the new type strain for the species (Dicks et al., 1996). Further research continued to call into question the phylogenetic relationship between the species using more refined methods such as comparative sequence analysis of the *recA* gene (Felis et al., 2001) and 23S-5S rRNA intergenic spacer regions (Chen et al., 2000). In 2002, it was proposed that the strain designated *L. casei* ssp. *casei* ATCC 393 should in fact be reclassified as *L. zeae* and that *L. paracasei* strains should be reclassified under *L. casei* ATCC 334 (Dellaglio et al., 2002). In 2008, the type strain of *L. casei* was confirmed to be the original strain ATCC 393, and the strain designated ATCC 334 was rejected as it represented a separate taxon, *L. paracasei* (Tindall, 2008). The revived name *L. zeae* was deemed to contravene Rules 51b (1) and (2) of the International Code of Nomenclature of Bacteria and has since been superseded by *L. casei* (Tindall, 2008). Current research continues to compare and reclassify known species within the group according to developed and novel methods.

## Difficulties in Speciation

The guidelines for probiotics in food according to the Food and Agriculture Organization of the United Nations and the World Health Organization (FAO/WHO) makes it mandatory that microorganisms which are used intentionally in food or animal feed must be taxonomically defined to genus and species level (FAO/WHO, 2002). As species of the LCG are present in a diverse range of habitats, and are used in the production of many food products in the food industry, molecular genotyping is crucial for determining their evolution and phylogeny. The LCG contains a high number of carbohydrate utilization gene clusters, allowing for survival and adaptation across a number of different environments (Cai et al., 2009). Traditional methods of characterisation for most species include phenotypic analysis such as carbohydrate fermentation profiles (Boyd et al., 2005), however, the gain and loss of plasmids can influence this phenotype and can make identification of strains problematic. Molecular genotyping through comparative analysis of the 16S rRNA gene is another widely used method of species identification (Weisburg et al., 1991). The 16S subunit of the bacterial ribosome is commonly used for species identification and differentiation as it is both ubiquitous and contains regions which are conserved among bacteria. Hyper-variable regions flanked by these conserved sequences allow for species identification based on sequence similarity, and has been used as the gold standard approach that avoids potentially confusing phenotypic identification methods (Petti et al., 2005). However, 16S gene analysis is not always reliable for LCG species due to high sequence similarities across the three species. Denaturing gradient gel electrophoresis (DGGE), which discriminates based on DNA fragment mobility under increasingly denaturing conditions, has been applied for the identification of LCG species, but has produced confusing results when compared with restriction endonuclease analysis of total chromosomal DNA (Vásquez et al., 2005). A comparison of randomly amplified polymorphic DNA (RAPD) analysis, ribotyping, and pulse field gel electrophoresis (PFGE) have all been applied for the differentiation of LCG species, but often yield contradictory results which lead to differing levels of speciation (Tynkkynen et al., 1999).

## Current and Future Trends

In recent years, other methods have been employed for the classification of LCG strains, **see** **Table 1**. A combined technique approach has been suggested to assist where conflicts occur in speciation (Iacumin et al., 2015). Using species-specific PCR and high-resolution melting (HRM) analysis on 201 strains from international culture collections belonging to the *L. casei* group, it was determined that 46 strains were different from their previous classification (Iacumin et al., 2015). A study was also conducted on 40 *L. casei* strains from plant material, human GITs, human blood, cheeses from different geographical regions, and unknown sources; these were characterized according to standard phenotypic methods (API biochemical testing) and multilocus sequence typing (MLST) using 6 housekeeping genes, together with PFGE (Cai et al., 2007). MLST revealed between 11 and 20 ‘allelic types’ based on the *nrdD* and *mutL* genes, and 36 sequence types. The study determined that PFGE was an effective method for discriminating isolates, even where MLST was unsuccessful (Cai et al., 2007). Another MLST study of 224 *L. casei* isolates from fermented foods using 10 housekeeping genes found no correlation with geographic location or food source, implying a complex evolutionary history and contradicting previous studies (Bao et al., 2016). Other single target genes used for species discrimination include housekeeping genes such as the chaperone protein gene, *dnaJ* (Huang et al., 2015), *dnaK* (Huang and Lee, 2011), *yycH* (Huang et al., 2014), and *mutL* (Bottari et al., 2017). Indeed, it was found that the *dnaJ* gene provided better resolution than the 16S rRNA subunit gene for comparisons within and between species, and this has been suggested as an alternative marker (Huang et al., 2015). The gene for the DNA mismatch repair protein *mutL* has been chosen as a target for species discrimination using multiplex PCR, and was found to provide a rapid method to distinguish *L. casei, L. rhamnosus* and *L. paracasei*, though in areas where conflicting results were found, whole genome sequencing (WGS) is suggested (Bottari et al., 2017).

**Table 1:** Recent publications differentiating species within the *L. casei* group.

Method of species differentiation/classification	# Strains	Results	Pros and cons to method	Reference
MALDI-TOF MS analysis of protein biomarker profile with proteomic analysis using in-house database	49	100% discrimination between *L. paracasei* subspecies. Discrepancies found between probiotic product descriptions and biomarker identification.	+ Rapid, accurate and cheap identification − Initial high cost of equipment − Requires database of known species biomarker profiles	Huang and Huang, 2018
Comparative genomics	183	Strains separated into three clades based on whole GC content, relatedness of conserved genes, and clustered based on functional genomic categories.	+ large number of comparison can be made with publicly available data − Potential difficulties with acquiring complete whole genomes of new strains	Wuyts et al., 2017
Multiplex PCR targeting the *mutL* gene	76	88% of strains identified, and improved characterization of 10 strains which couldn’t be identified by 16S.	+ Rapid method which can be used with relative ease (PCR based assay) + Can be used as a combined approach − Can provide some ambiguous results	Bottari et al., 2017
Species specific PCR and high-resolution melting (HRM) analysis	201	96.5% of strains identified as part of the LCG. 23.7% were in disagreement with species specific PCR.	+ Highly sensitive post- PCR technique	Iacumin et al., 2015
Phylogenetic analysis based on the *dnaJ* gene	30	*L. casei*, *L. paracasei* and *L. rhamnosus* separated, with better discrimination than the 16s rRNA gene.	+ Gene similarities of 81.7–85.5% at interspecies level allow for good separation − Requires specific kit and minisequencing technology	Huang et al., 2015
PCR amplification and comparison of *yycH* gene in LCG type strains	49	Strains were distinctly separated into clades according to species.	+ Average % gene identity of 78.5% among type strains allow for strong separation	Huang et al., 2014
Whole genome sequencing, annotation and comparative genomics	10	Differentiation of *L. casei* ATCC 393 from *L. paracasei* strains, and identification of conserved genomic islands and truncated genes in separate species.	+ In depth analysis of gene clusters − Limited number of species compared	Toh et al., 2013
Sequence comparison of the *dnaK* gene and RFLP	46	Strains were distinctly separated into clades per species.	+ Strong separation of species + PCR based method − Poor separation of subspecies	Huang and Lee, 2011

Whole genome sequencing has advantages in terms of the amount of sequence data provided, therefore allowing numerous gene target comparisons, and *in silico* DNA-DNA hybridisation (Stackebrandt et al., 2002). The ever decreasing cost of WGS is pushing current research in that direction. Numerous studies have used WGS as a method for distinguishing species of the LCG, though the methods are not without difficulty. Toh et al. (2013) produced complete genome sequences of *L. paracasei* JCM 8130 and *L. casei* ATCC 393, along with a draft sequence of *L. paracasei* COM0101 and a re-annotated *L. rhamnosus* ATCC 53103 (otherwise known as *L. rhamnosus* GG), and they compared them to other available sequences from LCG strains. Through comparative genome analysis, a core of 1,682 genes was found among the LCG and extensive genome wide synteny was observed, particularly for genomic islands containing carbohydrate utilization genes. The presence of truncated forms of the *spaCBA* pilus gene cluster previously identified in LGG was found in several *L. paracasei* strains (Toh et al., 2013). Such genomic insights are invaluable in determining evolutionary relationships within the group and may help differentiate strains where conventional methods fail. Comparative genomic analysis of 213 lactobacilli determined that the LCG clade harbor the most piliated species, which is unsurprising due to their well-known colonization of the GIT (Sun et al., 2015). Until now, such pan- and core-genome approaches have largely been ignored in taxonomy, though more studies are performing comparative genomics in order to determine evolutionary relationships, which in turn provide insights into taxonomy (Inglin et al., 2018). Pan-genomic approaches are highlighting inconsistencies in current taxonomy of the group (Wuyts et al., 2017), and comparative genomic studies have revealed high levels of intra-species diversity within the group, even among isolates from the same niche (Stefanovic and McAuliffe, 2018).

Isolates of *L. casei* have been found to have high levels of phenotypic and genotypic diversity, as has been demonstrated through fermentation profiling and MLST analysis (Cai et al., 2007). Comparative genome analysis of carbohydrate utilization clusters of *L. casei* strains found a loss of specific genes which enabled superior adaption for dairy isolates, particularly cheese strains, in the dairy niche, but decreased capacity to inhabit other niches when compared with *L. casei* ‘generalists’ as a result (Cai et al., 2009). In addition to gene decay among dairy isolates, comparative genome analysis of dairy, plant, and human strains found horizontal gene transfer from other LAB to be important in adaption of *L. casei* strains to new niches (Broadbent et al., 2012). *L. paracasei* strains have been found to display similar loss of genes in dairy isolates and high diversity among strains from other sources (Smokvina et al., 2013). It has been suggested that *L. rhamnosus* species have two distinct genotypes, one adapted to stable nutrient rich environments ie., milk and dairy products, and another which is more suitable to changeable environment such as the GIT (Douillard et al., 2013). A comparison of niche specificity across the LCG may be of interest in the future, in order to help unravel its taxonomic history.

Recently, novel methods have been developed using matrix-assisted laser desorption/ionization time-of-flight mass spectrometry (MALDI-TOF MS) to rapidly identify and classify bacterial isolates according to their protein profiles (Moore and Rosselló-Móra, 2011). This phenotypic proteomic approach has rapidly gained traction, and is being applied in laboratories (Neville et al., 2011), food (Pavlovic et al., 2013), and clinical settings (Samb-Ba et al., 2014). This method has been applied to differentiate species of the LCG after development of an in-house database of phenotypic profiles, and succeeded in identifying strains down to subspecies level (Huang and Huang, 2018). These methods are dependent on phenotypic protein profiles which may shift depending on culture conditions of the bacterium, and difficulties can occur due to initial high cost of equipment. Future approaches will likely depend on combined methods to assist in LCG species and subspecies differentiation, and without an agreed consensus on methods, the taxonomic future of LCG is likely to be as confusing as it’s past.

## The *L. casei* Group and Health Related Research

The LCG contains numerous strains with proven probiotic activities (Buriti and Saad, 2007). Probiotics are defined as live microorganisms that, when administered in an adequate amount, confer a health benefit on the host (FAO/WHO, 2001; Hill et al., 2014). Bacteria with associated health benefits have been researched since Elie Metchnikoff first suggested that the daily consumption of fermented milk products had a beneficial effect on human health (Ozen and Dinleyici, 2015). This research has been growing steadily over the last 100 years, with great strides having been made in linking health benefits with probiotic consumption. The work on these strains has progressed beyond the use of these bacteria in fermented dairy products, or even fermented foods in general, to a wider array of technological and medicinal purposes. As the mechanisms behind their health promoting capabilities are becoming unraveled, possible applications for these strains are being developed in the food, biotechnology and medical fields (Sanders et al., 2016). This is providing evidence for future novel non-dairy probiotic foods as well as potential for microbial-based therapies for certain disorders and diseases. Some of the more promising applications for the LCG in these fields will be discussed below. The ability of a number of these strains to help maintain a healthy microbiota may provide non-invasive adjunctive therapies for a range of disorders. They could be deployed prophylactically or therapeutically in a number of diseases linked to disturbances in the gut microbiota.

There is great potential in the fields of novel functional foods and pharmabiotics derived from the LCG. However, it should be noted that while many of these strains have been shown to have an effect in *in vitro* or *in vivo* mouse models, the underlying mechanisms require further research for any future use as microbial therapeutics.

**FIGURE 1:**
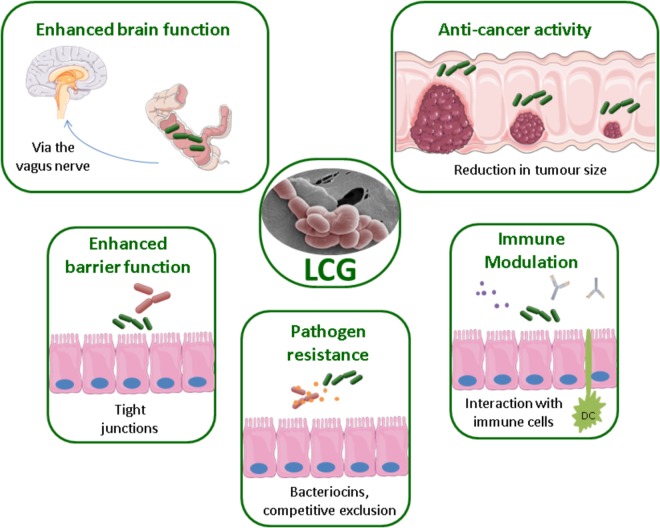
Methods of probiotic action by the LCG, EM of *L. paracasei* NFBC 338 (McGee et al., 2010).

Health benefits associated with the LCG have been reported for a variety of health conditions, ranging from atopic dermatitis to cancer. The mechanisms by which these bacteria directly or indirectly have a beneficial effect on human health are not yet fully understood and require further study. Potential mechanisms include the production of antimicrobial substances such as bacteriocins, enhancing the epithelial barrier through attachment, competition for pathogenic binding sites, or modulation of the immune system, see **Figure 1** (Bermudez-Brito et al., 2012). The processing and inclusion method of these strains has also been the subject of much interest. For example, the delivery matrix has been shown to influence the effects of *L. casei* BL23 in the treatment of colitis (Lee et al., 2015). Indeed, this strain only restored the microbiota when consumed in milk.

## Allergic Disease

Rates of childhood asthma are rising which has been linked to the hygiene hypothesis (Zhang et al., 2016). Essentially, this proposes that children are exposed to fewer microbes in infancy due to better hygiene, in addition to antibiotic exposure, birth by cesarean section and dietary changes. These factors influence development of the gut microbiota and its immune stimulation in early life. However, through modulation of the gut microbiota, probiotics have shown potential to reduce the risk of developing allergies such as atopic dermatitis. For example, the risk of developing allergy was shown to be reduced at 5 years of age by early life colonization with the LCG in children of both allergic and non-allergic parents (Johansson et al., 2011). The use of probiotics such as LGG have been investigated for their potential protective effect on children with a high risk of developing allergies such as asthma and atopic dermatitis (Kalliomaki et al., 2001). However, this study was recently repeated and found to be ineffective for the prevention of these allergies at 2 years (Cabana et al., 2017). A recent meta-analysis shows that there are confounding results in this area (Huang et al., 2017). While it is important to note this analysis included studies using multiple species and strains, it was concluded that the data overall indicate a benefit to the consumption of a probiotic, although this is dependent on many factors in addition to the specific strain used in the trials.

## Brain Function

A ‘psychobiotic’ is a bacterium which when administered in adequate amounts can have a positive mental health benefit (Dinan and Cryan, 2017). Members of the LCG have been involved in a number of important studies in this area. In one study focusing on the brain-gut-axis the strain *L. rhamnosus* JB1 highlighted both the ability of bacteria to positively affect brain function and also the importance of the vagus nerve in the bidirectional communication between gut microbes and the brain. Administration of JB1 directly affected the expression of GABA receptors in the brain which resulted in decreased anxiety and depressive behaviors in mice (Bravo et al., 2011). This study included healthy animals in separate laboratories with the same outcome.

In a recent study, a tablet preparation of LGG and *Bifidobacterium lactis* Bb12 as adjunctive therapy on hospitalized patients suffering from mania was effective in lowering rehospitalisation rates (Dickerson et al., 2018). While it is important to note this study had a number of limitations; the direct effect of the probiotic on the microbiome and inflammation of the CNS were not measured, as well as medication taken by the recruits was patient-specific. It does highlight the potential for microbial therapy in mental disorders which warrants further investment and study.

The ingestion of a combination of *L. rhamnosus* R0011 and *L. helveticus* R0052 also highlights the potential for bacteria to influence behavior in a mouse model. The study showed that modulation of the gut microbiota can positively influence certain animal behaviors, in this case stress-induced impaired memory (Gareau et al., 2011). The normalization of the gut microbiota through ingestion of this probiotic combination helped to prevent behavior abnormalities caused by infection with *Citrobacter rodentium*.

## Obesity

Obesity is a complex syndrome with a variety of contributing factors which influence onset. These include diet, physical activity and genetic factors, but also can be influenced by the composition of the gut microbiota. Manipulation of the microbiota through the administration of probiotics is an area that could provide interventions for the prevention and treatment of obesity. In a recent study, the probiotic *L. rhamnosus* CGMCC1.3724 was administered to obese men and women for 24 weeks. Males showed no significant difference in weight loss, but the female participants in the probiotic group had significant weight loss. This was found to be associated with reductions in blood leptin concentrations (Sanchez et al., 2014). Obesity is also associated with changes in the gut microbiota and immune parameters. A fermented milk containing *L. casei* CRL 431 fed to mice was able to positively affect the microbiota as well as some biomarkers associated with obesity (Nunez et al., 2014).

In a murine model of obesity *L. casei* Shirota was evaluated for its ability to improve weight management in comparison to Orlistat, a drug used to treat obesity (Karimi et al., 2015). The results of this study showed promise in the use of probiotic strains as an alternative method for weight management. The impact of exposure to probiotics on weight gain before birth has also been investigated. *L. rhamnosus* administered to pregnant women has been found to modify weight gain in children during the first 6 months of life (Luoto et al., 2010).

## Cancer

Cancer is a global concern with large efforts being devoted to methods to prevent and/or cure the disease. Cancer is the abnormal growth of cells beyond their usual boundaries that can invade adjoining parts of the body and/or spread to other organs (WHO, 2015). Probiotics have been investigated for their potential as an adjunctive therapy and as a microbial therapy for cancer (Huang et al., 2016; So et al., 2017).

The previously mentioned *L. casei* species type strain ATCC 393 was investigated in an experimental model of colon cancer using both murine and human colon carcinoma cell lines (Tiptiri-Kourpeti et al., 2016). The *in vivo* model showed an approximately 80% reduction in tumor volume in mice fed live *L. casei* for 13 days. The bacteria attached to the cancer cells and reduced cancer cell viability and induced apoptotic cell death. While the mechanisms are not fully understood, this provides promising evidence for the use of *L. casei* in the treatment of cancer. *L. casei* strains have also been investigated for prophylactic treatment in late stage colorectal cancer (CRC). The main cause of death from CRC is the tumor metastasising to other organs. Cell free supernatant from *L. casei* and *L. rhamnosus* was found to lower the ability of a metastatic tumor cell line to invade *in vitro* (Escamilla et al., 2012).

Numerous studies have investigated strategies for how probiotic strains may be used to aid tumor reduction. *L. casei* Shirota has been studied in combination with dietary fiber for its ability to reduce the reoccurrence of tumors in colorectal cancer, with early studies showing promising results (Ishikawa et al., 2005). LGG has also been investigated as a synbiotic with oligofructose and *Bifidobacterium lactis* Bb12 (Rafter et al., 2007). This promising study showed that the synbiotic was capable of lowering the uncontrolled growth of intestinal cells. This is thought to be due to presence of the synbiotic enhancing the mucosal structure, reducing exposure of epithelial cells to cytotoxic and genotoxic agents, and a decrease in colonic cell growth.

Treatment methods for cancer patients can put a large burden on the immune system and can cause side effects such as diarrhea which, in an immunocompromised individual, can prove fatal. Probiotics have been shown to reduce the likelihood of radiation-induced diarrhea during cancer treatment (Liu et al., 2017). LGG has also been studied as an adjunctive therapy for cancer treatments to reduce the incidence and severity of diarrhea (Banna et al., 2017). It was shown that treatment supplemented with LGG capsules resulted in less grade 3/4 diarrhea, with less abdominal discomfort reported (Osterlund et al., 2007).

## Diarrhea

According to the WHO, diarrhea is the second highest cause of death in children under 5 years old, and remains a major problem associated with antibiotics. Antibiotic-associated *Clostridium difficile* infection is the leading cause of diarrhea in high-income countries at all ages. LCG strains have been associated with improving the symptoms and/or duration of diarrhea in numerous studies, but specifically LGG is a promising strain in this regard. The ability of many *L. casei* strains to prevent antibiotic associated diarrhea is associated with its ability to maintain the diversity of the gut microbiome of individuals during antibiotic treatments. This could be due to direct attachment of the strain to epithelial cells. LGG attaches to mucosal cells through the SpaCBA pilus. The SpaC adhesin is present along the entire length of the pilus in large quantities (Reunanen et al., 2012). This adhesin allows both long and close adhesion of the bacterium and aids in the long term colonization of the strain. Vancomycin resistant enterococci, such as *Enterococcus faecium* also bind to and colonize the gut based on a pilus with high sequence similarity to the LGG SpaCBA pilus. LGG could be used prophylactically or as direct competition in the treatment of enterococcal infections (Tytgat et al., 2016).

## Microbial Therapy Through Bacterial Components

The future of probiotics lies not only in the administration of live bacteria but also in the possibility of utilizing bacterial components to modulate the immune system. The ability of *L. casei* strains to survive passage through the digestive system and potentially colonize the gut allows some strains to have direct effects on human health. However, bacterial components are also being investigated for their health associated capabilities. In one study, a heat-killed strain of *L. casei* was administered in a mouse model of colitis. Both the heat-killed and live strains were shown to have therapeutic effects on a model of inflammatory bowel disease (IBD) (Thakur et al., 2016). The advantage of such a scenario is promising as it eliminates the need for the strain to survive passage through the gut and could potentially provide a more consistent effect as colonization is not required.

Exopolysaccharides (EPS) are produced by numerous bacteria, sometimes in response to stress. EPS have also been investigated for their health-promoting benefits (Ryan et al., 2015; Caggianiello et al., 2016), such as immunoregulatory effects (Laino et al., 2016). The LCG have long been used in fermentations whether as adjunct or starter cultures. Traditionally these would have been included for their desirable technological properties such as EPS production. Many of these technologically desirable traits have since been investigated for their probiotic potential. For example, EPS produced by *L. rhamnosus* has been shown to have immunosuppressive capabilities (Bleau et al., 2010). The probiotic strain *L. paracasei* DG has a range of health promoting properties attributed to it, including its use as an adjunctive therapy in *Helicobacter pylori* treatment and modulation of the gut microbiota. Recent suggestions that bacterial components may be the mechanism by which probiotics exert their effects sparked a study on the EPS produced by this strain. This study demonstrated that the EPS which is secreted by and covers *L. paracasei* DG may have a role in its health associated properties (Balzaretti et al., 2017).

Interest in utilizing probiotics for the treatment or prevention of disease is growing, and the studies discussed here highlight some of the potential directions for the future of health related research in the LCG. The LCG exert many of their health benefits when ingested live and are capable of colonizing the gut. To survive this transit they must have the propensity to respond to a wide array of stresses. The underlying mechanisms involved in this are becoming clearer.

## The *L. casei* Group and Stress Response

As probiotics the LCG must endure many stresses during processing and remain viable on transit to their site of action, the GIT. These stresses include but are not limited to bile salts, oxidative stress, cold stress, osmotic stress, acid stress and long term storage. Resistance to these stresses in LCG is strain dependent, with some having a high resistance to multiple stresses while others confer little to no resistance (Reale et al., 2015). The LCG employ many different tactics in order to survive these stresses, including alterations to cell membrane, metabolic pathways, and upregulation of chaperone proteins (Maria and Marco, 2004).

## Acid Shock

Due to the facultative heterofermentative nature of LCG, hexose sugars are almost exclusively fermented to lactic acid. The LCG have an innate tolerance to acid stress for this reason. Probiotic bacteria must also face an acidic environment during gastrointestinal transit. Tolerance to acid stress is particularly relevant in industry for probiotic bacteria. In acidic environments, acids can passively diffuse into cells where they can rapidly dissociate into protons. This lowers the intracellular pH (pHi) and affects the transmembrane pH gradient and the proton motive force (van de Guchte et al., 2002; Corcoran et al., 2008; Mills et al., 2011). Low pHi also causes damages to proteins and DNA. The methods used to counteract acid stress in *L. casei* include the Arginine deiminase pathway, F0F1 proton pump, cell membrane alterations and repair of damaged DNA and proteins. Amino acid utilization also plays a physiological role in pHi control in LAB (Fernández and Zúñiga, 2006).

The LCG can exploit amino acids such as arginine, histidine, and aspartate during acidic conditions (Broadbent et al., 2010; Wu et al., 2012a; Wu et al., 2013). This is generally achieved by deamination of the amino acid and the expulsion of the alkaline product from the cell (Corcoran et al., 2008; Papadimitriou et al., 2016). The arginine deiminase pathway is a catabolic system found in *L. casei* that uses arginine to produce ATP, ornithine CO_2_ and NH_3_ (Marquis et al., 1987). The system is comprised of three enzymes; arginine deiminase (ADI), catabolic ornithine transcarbamoylase and carbamate kinase, and a singular membrane transport protein (Papadimitriou et al., 2016). The NH_3_ reacts with protons to help alkalize the extracellular environment. Acid resistant *L. casei* can overexpress proteins involved with the ADI pathway in acidic conditions (Wu et al., 2012a). The F0F1-ATPase is ubiquitous among bacteria (van de Guchte et al., 2002). This multicomponent enzyme is bifunctional; it can produce ATP using protons or alternately pump protons out of the cell by hydrolysing ATP (van de Guchte et al., 2002). In acidic conditions, acid tolerant *L. casei* show higher levels of ATPase activity than that of acid sensitive cells (Wu et al., 2012a). Bender and Marquis showed that *L. casei* had a higher ATPase activity that that of the non-acid tolerant *Actinomyces viscous* in acidic conditions (Bender and Marquis, 1987).

Bacteria also adapt to environmental changes (stresses) by modifying their membrane composition (Zhang and Rock, 2008). The acid tolerant *L. casei* Zhang can increase expression of the proteins MurA and MurG, key enzymes in peptidoglycan synthesis (Wu et al., 2012a). It has been shown that bacteria alter the fatty acid composition of their cytoplasmic membrane in response to acidic conditions but different results have also been reported. Fozo et al. (2004) and Wu et al. (2012b) reported that there is an increase in the unsaturated fat proportions while Broadbent et al. (2010) have reported the opposite in that the ratio of unsaturated fatty acids increases. *L. johnsonii* has been shown to become more sensitive to acid stress when supplemented with unsaturated fatty (Muller et al., 2011). These contradicting results could be due to the use of different strains or different experimental conditions. In response to DNA damage, there is an overproduction of DNA repair proteins involved in base excision repair, nucleotide excision repair, mismatch repair, and homologous recombination (Wu et al., 2012a). Proteins involved in the general stress response (DnaK, DnaJ and Hspl) and chaperone proteins (GroEL, GrpE) are also overexpressed (Wu et al., 2011, 2012a, 2014). These proteins are involved in the prevention of incorrect protein folding and repair of damaged proteins at the expense of ATP (Schröder et al., 1993). Many of the reactions of the cell to acid stress are energy dependent and therefore require ATP. Wu et al. (2012a) found that while levels of glucose-phosphotransferase system (PTS) activity dropped significantly in acid stressed cells, they were still higher than in that in sensitive cells. This in turn means more glucose entering glycolysis and more energy (ATP) for the cell to react to acid stress. Nezhad et al. (2012) also showed that there were increased levels of proteins involved with the glycolytic pathway expressed on the cell surface of *L. casei* under acidic conditions.

## Cold Shock

Post fermentation, probiotic LCG are going to encounter cold stress in the form of refrigeration. Survival of cold storage is necessary for refrigerated probiotic bacteria to reach their final destination in the human GIT. Cold is a physical stress that affects physico-chemical properties inside cells. It does this by influencing membrane fluidity, diffusion rates and interactions of macromolecules such as proteins, DNA and RNA. Cold shock proteins (CSPs) are produced by bacteria in response to growth at temperatures below optimum conditions. CspA and CspB in *L. casei* have similar sequences to that of the C-terminal of EIIA proteins of the glucose-PTS system (Monedero et al., 2006). It has been suggested that Hpr phosphorylates these cold shock proteins to activate them in response to cold shock (Beaufils et al., 2007). *L. casei* mutants unable to form Hpr ser-45 were much more sensitive to freeze/thaw when grown on glucose as compared to growth on other sugars (Beaufils et al., 2007). This suggests that CSPs have a major role in metabolism at low temperatures. More cold-induced proteins (CIPs) are expressed in cold shock to maintain fluidity of the cell membrane by increasing the ratio of shorter and/or unsaturated fatty acids and to maintain DNA structure by reducing negative supercoiling.

## Bile Stress

Bile acids (sometimes referred to as salts) are secreted into the intestine of mammals to aid digestion. They also offer another hurdle for bacteria as they have antimicrobial activity. Bile is known to affect the structure of membranes, DNA, RNA and protein folding (Corcoran et al., 2008). Hamon et al. (2012) employed a proteomic method to evaluate bile tolerance in *L. casei* and suggested the membrane modification protein RmlC, cell wall synthesis protein NagA and NagB, molecular chaperone protein ClpP and proteins involved in central metabolism were key to bile tolerance. Bile salt hydrolase (BSH) is a protein expressed by bacteria commonly found in the intestinal tract that can deconjugate bile salts and also have the added positive effect of lowering serum cholesterol levels and reducing the risk of obesity and arthrosclerosis in the host (Wang et al., 2011). BSH activity has been found in *L. casei* but is not always present (Gilliland and Speck, 1977; Zhang et al., 2009; Wang et al., 2011). There is conflicting evidence whether or not BSH increases tolerance to bile salts with many studies finding a correlation (Moser and Savage, 2001; Wang et al., 2011) while many others have failed to find correlation between BSH activity and bile tolerance (Begley et al., 2005; Bustos et al., 2012).

## Oxidative Stress

Oxidative stress refers to the production of Reactive Oxygen Species (ROS) and accumulation in the cell. ROS include superoxide (O_2_-), hydrogen peroxide (H_2_O_2_), and hydroxyl radical (OH). ROS can lead to impairment of proteins, lipids, and nucleotides, contributing to the arrest of cell growth and cell death (Imlay, 2003). Some LAB, including *L. casei*, can utilize oxygen in aerobic conditions for energy production using a pathway which includes pyruvate oxidase, NADH oxidase, NADH peroxidase and acetone kinase, which results in the formation of ROS (Maria and Marco, 2004; Ianniello et al., 2016). Catalase is an enzyme capable of degrading H_2_O_2_ and therefore is a key component in oxidative stress. Indeed, the presence of oxygen has been shown to increase its activity in *L. casei* (Ianniello et al., 2016). The high Mn content of *L. casei* can act as an efficient scavenger of O_2_, thereby compensating for the lack of a superoxide dismutase, while there are currently no known enzymes to degrade hydroxyl radicals (van de Guchte et al., 2002).

## Osmotic Stress

High osmolality is often faced by the LCG in fermented foods. Food spoilage bacteria and pathogens are often more sensitive to high osmotic conditions compared to LAB, therefore NaCl is often added to aid indigenous or starter LAB during the fermentation process (Papadimitriou et al., 2016). Salt stress leads to cell wall conformational changes in *L. casei*, which increases its ability to form biofilms and bind cations (Palomino et al., 2013). High salt conditions cause *L. casei* to become more susceptible to antimicrobial peptides which target cell wall synthesis (Piuri et al., 2005; Palomino et al., 2013).

## Storage

Before use as a probiotic, bacteria may need to be stored for long periods of time. The two main methods for preparing them for long term storage are freeze-drying and spray-drying. During freeze drying, cells are subjected to a physical stress caused by crystals forming, dehydration and osmotic stress (Broeckx et al., 2016). Jofré et al. (2015) found members of the LCG survive storage better at refrigeration temperature and skimmed milk alone or supplemented with trehalose or lactose to be a suitable cryoprotectant during freeze-drying. Encapsulation of *L. casei* cells during freeze drying can also offer protection to cells during storage post freeze drying (Xu et al., 2016).

During spray drying, bacteria are exposed to many different types of stress which affect the viability of the cells during storage including heat, dehydration, shear, osmotic and oxidative stress (Broeckx et al., 2016). Microencapsulation of the cells in a protective agent is one of the main methods of increasing viability during spray drying. *L. casei* LK-1 microencapsulated in skimmed milk showed better survivability during storage and gastric digestion than cells microencapsulated in trehalose and maltodextrin (Liao et al., 2017). Storage temperature is also a key factor in the viability of cells following this process. An increase of storage temperature from −20°C to 4°C or from 4°C to 25°C causes a three fold acceleration of the inactivation rate of *L. casei* LK (Liao et al., 2017). Higher inactivation rates for *L. rhamnosus* GG were also reported at room temp following spray drying compared to chilled storage temperatures (Soukoulis et al., 2016).

## Conclusion

The LCG contains the species *L. rhamnosus*, *L. casei*, and *L. paracasei*; these are well-researched due to their applicability in the food, biopharmaceutical and medical industries. Despite this, the group has a long complicated taxonomic history in distinguishing and discriminating the species from each another. The taxonomic debate surrounding the LCG is likely to continue to prove interesting and challenging as sequencing and novel identification methods are developed.

Stress resistance is associated with the LCG through physical and metabolic alterations; although these resistant phenotypes are strain specific. The LCG must be capable to survive a number of stress conditions if they are to be used in industry, including oxidative stress, osmotic stress, cold stress, acid stress and long term storage. In addition to their need to tolerate these conditions in food processing, live strain must be able to survive passage through the GIT if intended for health-promoting purposes.

The health-promoting capabilities of the LCG have been documented in several studies suggesting real potential for their use in the treatment, or prevention, of a variety of diseases. Moving forward, it is essential that scientists decipher the underlying mechanisms of action involved in order to be able to apply these strains or their bacterial components as novel treatments or prophylactic interventions.

## Author Contributions

DH, IS, and CT contributed equally to the writing of the manuscript. CH, CS, and RR contributed to manuscript revision, read, and approved the submission.

## Conflict of Interest Statement

The authors declare that the research was conducted in the absence of any commercial or financial relationships that could be construed as a potential conflict of interest.
